# Field Monitoring of Column Shortenings in a High-Rise Building during Construction

**DOI:** 10.3390/s131114321

**Published:** 2013-10-24

**Authors:** Se Woon Choi, Yousok Kim, Jong Moon Kim, Hyo Seon Park

**Affiliations:** 1 Department of Architectural Engineering, Yonsei University, Seoul 120-749, Korea; E-Mails: watercloud@yonsei.ac.kr (S.W.C.); yskim1220@yonsei.ac.kr (Y.K.); 2 Dasumtek Incorporated, Seoul 222-22, Korea; E-Mail: dstek@dstek.biz

**Keywords:** high-rise building, column shortening, wireless sensor network, automatic measurement

## Abstract

The automatic monitoring of shortenings of vertical members in high-rise buildings under construction is a challenging issue in the high-rise building construction field. In this study, a practical system for monitoring column shortening in a high-rise building under construction is presented. The proposed monitoring system comprises the following components: (1) a wireless sensing system and (2) the corresponding monitoring software. The wireless sensing system comprises the sensors and energy-efficient wireless sensing units (sensor nodes, master nodes, and repeater nodes), which automate the processes for measuring the strains of vertical members and transmitting the measured data to the remote server. The monitoring software enables construction administrators to monitor real-time data collected by the server via an Internet connection. The proposed monitoring system is applied to actual 66-floor and 72-floor high-rise buildings under construction. The system enables automatic and real-time measurements of the shortening of vertical members, which can result in more precise construction.

## 1. Introduction

Vertical members in a high-rise building, such as columns and shear walls, receive vertical loads that subsequently lead to axial shortenings of the members. Factors that influence the extent of the shortening of a vertical member include the stress ratio induced in the member during construction, the reinforcement ratio of the section, and other environmental conditions surrounding the member [1]. The vertical members in a high-rise building will not shorten by the same amount since the vertical members have different loading and design conditions, so differences between the amount of shortening of adjacent vertical components inevitably occur.

This differential shortening can deteriorate the quality of construction by causing slab distortions, separation of elevator guiderail brackets, unexpected deformation of interior drywalls, and damage to cladding systems [2,3]. In practice, to minimize the effect of differential shortening on structural and nonstructural elements, compensation for the estimated differential shortenings is made during construction [1]. Thus, during construction, to compensate for differential shortening, the formwork along one edge may be raised by the estimated amount of the differential shortening. However, it is almost impossible to accurately predict the amount of shortening through an estimation formula due to the idealization of material properties and the assumptions in analytical models [4–6].

**Figure 1.**
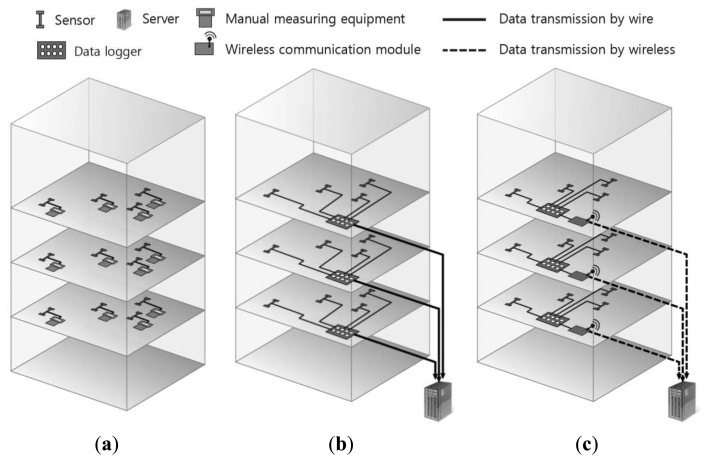
Existing systems for the measurement of column shortenings in a high-rise building. (**a**) Manual measurement; (**b**) Wired measurement; (**c**) Wireless measurement.

Therefore, a field monitoring program is used to obtain actual shortenings of walls and columns and to compare them with the estimated shortenings. For a proper compensation of differential shortening [7,8], the comparison between measured and estimated shortenings is made at selected floor levels, where strain sensors are installed, throughout the height of the building. There have been many cases in which the amount of shortening was measured in the process of constructing actual high-rise buildings [2,9–13]. As shown in Figure 1, conventional field sensing systems for measurement of column shortenings during construction may fall into three categories. One method, for manual data acquisition, is to use a manual instrument to take direct measurements from each sensor [13]. A second method, for wired data acquisition, is to install a data logger on each floor where sensors are installed, connect the data logger and sensors with a cable, and connect this to the server for automatic measurement. The third method, for wireless data acquisition with a conventional data logger, is to connect the sensors and a conventional data logger with lengthy cables and then connect this to a wireless communication device to send to the server [12,14]. In practice, the most widely used practice is to connect a local area network (LAN) or wireless communication device to the data logger to collect data automatically, such as in the second and third method.

However, these methods necessitate connecting the sensors on each floor to the data logger with cables or wires, which can disturb the construction process and cause significant time and effort for management of lengthy cables. Additionally, no research on the automatic monitoring system for shortenings of vertical members in high-rise buildings during construction, which can confirm the data collected in the server in real-time for the prompt management as well as measure the shortenings automatically, has been reported. Therefore, to solve the problems associated with cables during construction and monitor the collected data in real-time, an automatic field monitoring system using a wireless sensor network (WSN) is necessary [15–17]. An automatic field monitoring system based on a WSN is convenient to install and manage during construction. Also, a lot of data can easily be measured from numerous sensors, and the extent of column shortenings in a high-rise building can easily be analyzed according to the construction process. This can enhance the precision and safety of the construction of a high-rise building.

In this paper, an automatic field monitoring system to measure the shortening of column and walls in high-rise buildings during construction is presented. This system comprises the following components: (1) a WSN that can automatically measure vertical shortening using numerous sensors embedded in columns and walls and transmit the measured data to a server and (2) monitoring software that enables construction administrators to monitor the real-time data collected on a server via an Internet connection. To improve the applicability of long-term monitoring of the shortening of vertical members in high-rise buildings under construction, wireless sensing units (sensor nodes, master nodes, and repeater nodes) with power-saving functions and acceptable diffraction capabilities are employed in the WSN. The proposed monitoring system can contribute both to the prompt verification of collected data from any location and to the automatic sensing of the shortening of vertical members in high-rise buildings under construction. The proposed system was applied to the construction sites of actual 66-floor and 72-floor high-rise buildings to monitor shortenings during construction. 39 vibrating wire strain gauge sensors, 23 sensor nodes, and four master nodes were installed in the automatic sensing system. The measurement data collected by the proposed monitoring system during the monitoring period of 88 days are provided and discussed.

## 2. WSN for Monitoring of Column Shortening in High-Rise Buildings

**Figure 2.**
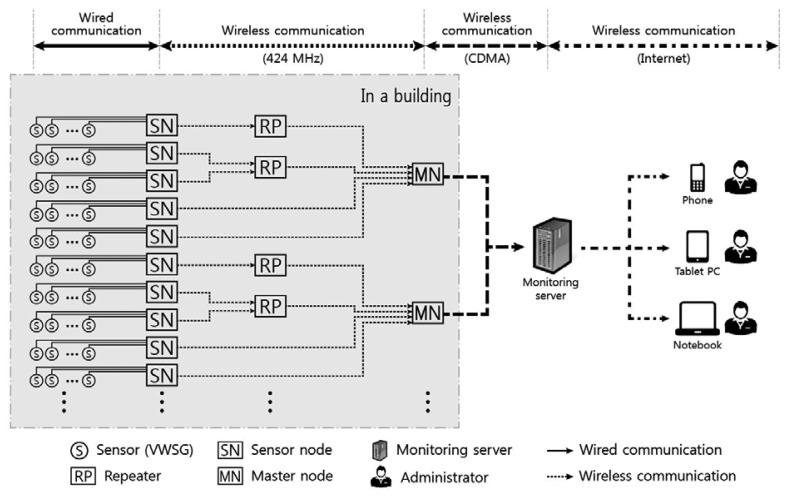
Wireless sensor network system for measurement of column shortenings in a high-rise building.

The wireless sensor network system for field monitoring of vertical shortenings of columns and walls in a high-rise building is presented in Figure 2. The system comprises a WSN installed within the building as well as a server that stores and manages in real-time the transmitted data. The WSN system comprises the sensors, sensor nodes, master nodes, and repeater nodes. Sensor nodes are directly connected by wires to sensors to take automatic measurements of shortenings in vertical members, which are then transmitted wirelessly to the master node. The master node's function is to wirelessly transmit data collected from numerous sensor nodes to the server. When there is a problem in communication between sensor nodes and the master node due to various obstacles such as dry-walls, repeater nodes are installed between sensor nodes and the master node to solve the communication problem. This WSN system minimizes the amount of cables needed to be installed and automates the processes of data measurement, collection, and transmission. The data collected through the WSN are transmitted to the server and can be monitored in real-time through a web-based managing program. In the following sections, the characteristics of each component of the automatic measurement system are explained.

### 2.1. Wireless Sensor Network (WSN) System

#### 2.1.1. Sensor

**Figure 3.**
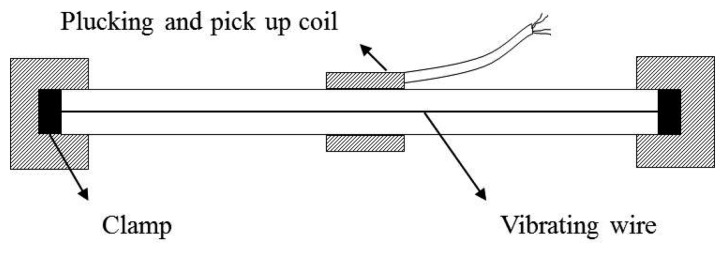
Vibrating wire strain gauge.

Generally, vibrating wire strain gauges (VWSGs) have been widely used to measure axial shortenings of columns and walls during construction of high-rise buildings since VWSGs are not subject to electromagnetic interference (EMI) and have excellent endurance properties [9,10,13]. As shown in Figure 3, a VWSG [18] basically consists of two major components: a vibrating wire whose frequency changes in response to tension or compression and two mounting blocks which are fixed at the ends of the vibrating wire, where the mounting blocks are induced as tension or compression in structures. In Figure 3, a plucking coil excites the vibrating wire and measures resonance frequency of it. A change in the length of the vibrating wire due to tension or compression leads to variation of the natural frequency of the wire. For a given pretension of the wire during the initial setup, the shortening of a member is calculated from the relationship between the natural frequency of a vibrating wire and the strain of the wire [18,19].

#### 2.1.2. Sensor Node

The existing technology for measurement of column shortenings generally involves installing a single data-logger on the floor where the sensors are installed and connecting all of the sensors to the logger in a given floor with cables. For the case of a high-rise, the cables connecting sensors to the logger can be several meters in length. To solve the problems associated with cables during construction, it is necessary to minimize the length of cable between the sensor and the data-logger, and automate the monitoring process, including data measurement, collection, and analysis.

**Figure 4.**
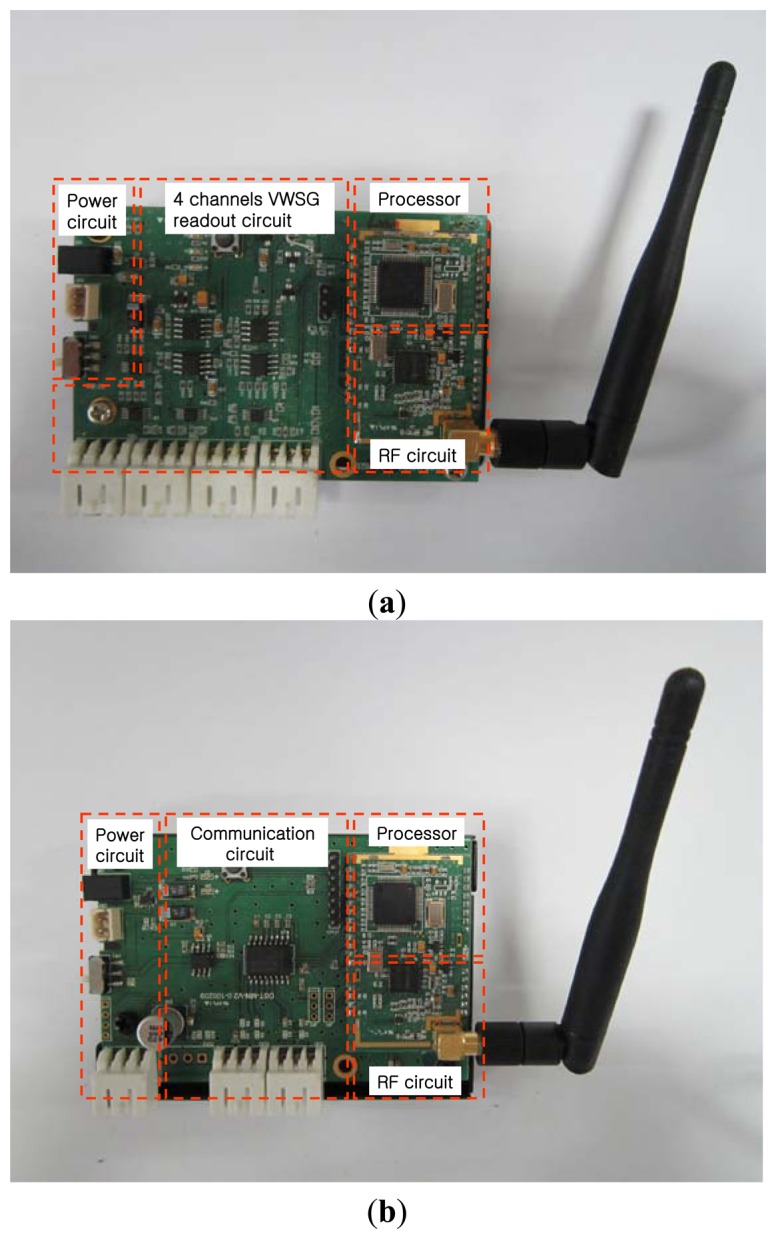
Wireless sensor node and master node. (**a**) Sensor node; (**b**) Master node.

In this paper, to avoid lengthy cables between the logger and sensors, a VWSG-based wireless sensor node is used in measurement of column shortenings [19]. As can be seen in Figure 4a, the sensor node comprises a four channel VWS sensor module, a wireless communication module, and a processor and power-saving circuit. The sensor module excites the vibrating wire of the sensor and reads the resonance frequency from the sensor. To reduce the number of sensor nodes installed in high-rise buildings, this can simultaneously measure a maximum of four sensors using “demuxing” and “muxing” of signals. In buildings and civil infra-structures, a WSN using UHF or microwave frequency is generally employed [20–22]. The wireless communication module used in this study was the Industrial Scientific and Medical (ISM) frequency band at 424 MHz UHF, which has better diffraction, less electricity consumption, and less influence from signal disturbance than microwaves. This has valid communication distances of 300 m and within 70 m for line of sight (LOS) and non-line of sight (NLOS), respectively.

The processor minimizes power consumption by controlling the operation of sensor nodes using the built-in memory program. In other words, the timer in the processor causes alternation between “action mode” and “sleep mode”. In action mode, strains are measured and transmitted to master node or repeater node using a wireless communication module. Then, when the sensor node changes to sleep mode, the power is changed to an energy-saving mode.

#### 2.1.3. Master Node and Repeater Node

The master node shown in Figure 4b comprises a short-distance wireless communication device (low-power wireless modem of ISM band) and a long-distance communication device (Code Division Multiple Access—CDMA). As shown in Figure 2, the master node receives measurement values through short-distance communication with sensor nodes installed in the building and transmits the collected data to the server outside of the building through long-distance communication. CDMA used in master node does not have distance limits, as a specific user's coded signal appears as noise to other users, and there is a low possibility of data noise from long-distance transmission. Therefore, there are fewer limitations to the position of the server receiving data, meaning it can be installed far from the site.

The repeater node comprises only a short-distance wireless communication device. Thus, it is used in cases where communication between sensor node and master node is not smooth. A repeater node deciphers the communication between master node and sensor node by differentiating the frequency channel. According to the specific circumstances, there may be one or more repeater nodes between the sensor node and master node.

#### 2.1.4. Wireless Sensor Network (WSN)

As can be seen in Figure 2, WSN is based on a one-tier topology, sending collected data from sensor nodes to master node. However, communication problems can result from obstacles, including both structural and non-structural components within the building, and therefore, a tree topology can be employed at locations where repeater nodes are installed to transmit data between the sensor node and the master node. The tree topology is extensively applied in SHM to improve the limitation of one-tier topology, such as the power consumption and range of wireless communication [23–25]. Data can be collected through bidirectional communication between sensor nodes and master nodes; however, this communication can consume a substantial amount of power compared with unidirectional communication. The power consumption is a very important issue for the long-term automatic monitoring of buildings.

Therefore, this study uses unidirectional communication in which data are transmitted from sensor nodes to a master node. In the case of unidirectional communication, the master node and repeater node must always be on standby in order to receive data since it is not known when the sensor nodes will send data. This requires a large amount of energy consumption. This problem can be solved by repeating the sleep-action process, activating it when data collection is required and subsequently changing it back to sleep mode. The wireless nodes in this system, including sensor nodes, repeater nodes, and master nodes, possess the power-saving capability of alternating between sleep mode and action mode. The sleep-action mode for each wireless node is independently repeated to minimize power consumption. However, the independent operation of each wireless node results in unavoidable data loss. To simultaneously consider data loss and power consumption in this study, the sensor nodes are set to perform the action mode more frequently than are the master nodes. For example, if the sensor nodes transmit data to the master nodes at intervals of thirty minutes and the master nodes are operational for one hour and are then turned off for the next three hours, the master nodes can receive a minimum of four measurement data from the sensor nodes per day. A small 2,700 mAh battery can be used for a minimum of 200 days if 48 measurements are performed each day.

### 2.2. Sever and Monitoring Software

All data measured by the sensors installed at the site are transmitted to the server through the WSN system. This server can be located either at the construction site or off-site, where it can simultaneously receive and manage data transmitted from numerous construction sites.

The server comprises data reception module, database module, and situational awareness module. The data reception module receives data sent by the master node through a CDMA modem and transmits it to a database module. The database module is where all the information handled by the web-based management system, as well as the data transmitted by master node, are stored. This is also where the various internal procedures related to data storage and program operations are registered. Through the database module, the managing program can conduct data queries and the data can be stored and managed according to the type. The situational awareness module analyzes received data regularly to evaluate structural safety and risk. When the level of measured structural response is larger than the established acceptance criteria, the module sends a message through Short Message Service (SMS), e-mail, *etc*. The situational awareness module also has a regular analysis and alarm function regarding the condition of sensors and nodes so that action can be taken promptly.

As shown in Figure 2, the server can be connected, anywhere and anytime, to check the situation at the site in real-time through the web-based managing program. Because the data received by the server is accessible online, the situation at the site can be monitored in real-time without the necessity of further action. For the convenience of site management, the server can also manage several sites where wireless sensor systems are installed and many users can connect to the server simultaneously.

## 3. Field Application

### 3.1. Building Outline

**Figure 5.**
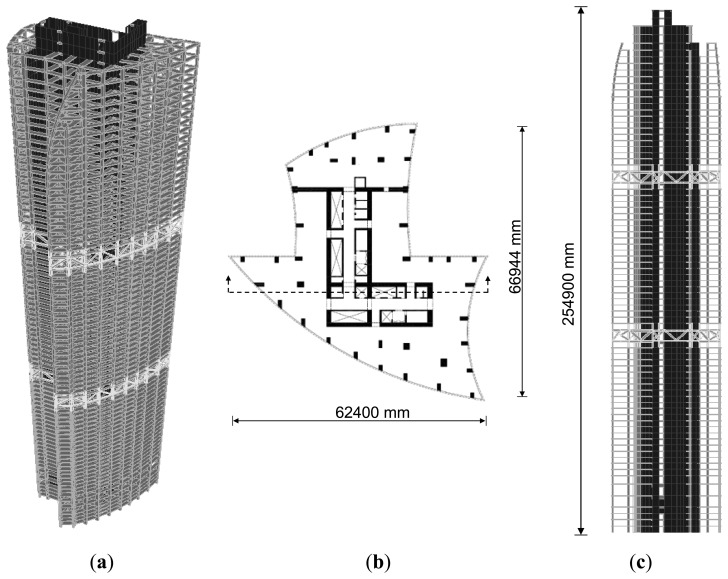
72-story high-rise building (Tower 2). (**a**) Perspective view; (**b**) Typical floor plan; (**c**) Vertical section.

The proposed system was applied to the construction site of actual high-rise buildings to be used for residential purposes as shown in Figure 5. The buildings are composed of two towers (tower 1: 66 stories; tower 2: 72 stories). A typical floor plan for the towers is shown in Figure 5b. Reinforced concrete shear walls, outriggers, and belt trusses were used as the lateral load resisting system for the towers (Figure 5c), and the exterior columns were designed to be steel reinforced concrete columns.

### 3.2. Installation of Sensors and Wireless Nodes

**Figure 6.**
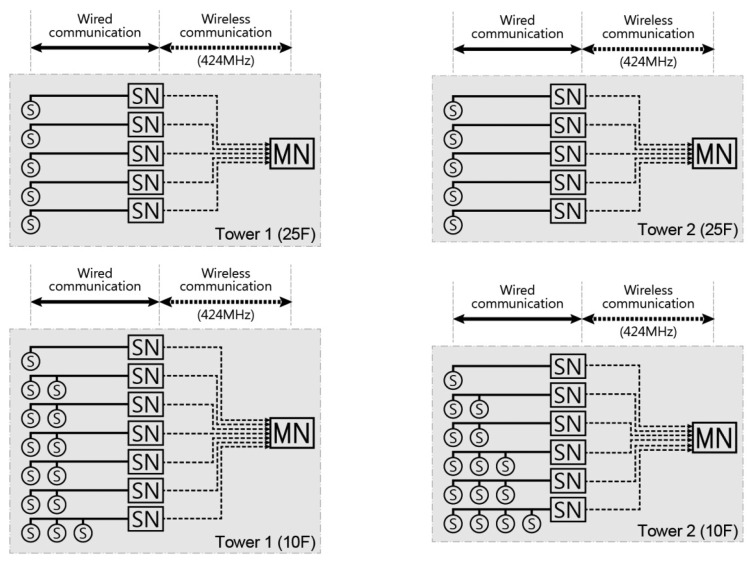
Conceptual diagram of WSNs for tower 1 and tower 2.

**Figure 7.**
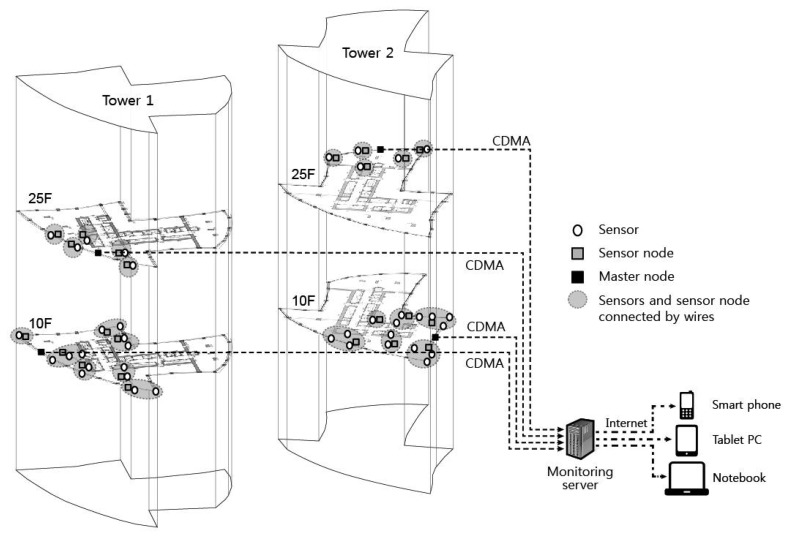
Location of sensor nodes and master nodes for field measurement of column shortenings in tower 1 and tower 2.

The location of sensors to be installed is generally planned in consideration of the floor plan and elevation of the structure. The basic principle of elevation plan is to select floors from which long-term monitoring of shortening is possible. In this application, sensors were installed on the 10th and 25th floors in tower 1 and on the 10th and 25th floors in tower 2, as shown in Figure 6. The number of installed sensors and nodes is shown in Table 1. Taking into account the locations of the sensors and partitions that could obstruct wireless communication, the locations of sensor nodes and master nodes for both towers are shown in Figure 7. Sensor nodes, which were connected by short wires to sensors, automatically collected measured data and sent the information to master node installed on the same floor through wireless communication. Master node sent the data from numerous sensor nodes to the server through long-distance wireless communication. The data stored in the server were available in real-time from any place with an internet connection through a web-based managing program.

**Figure 8.**
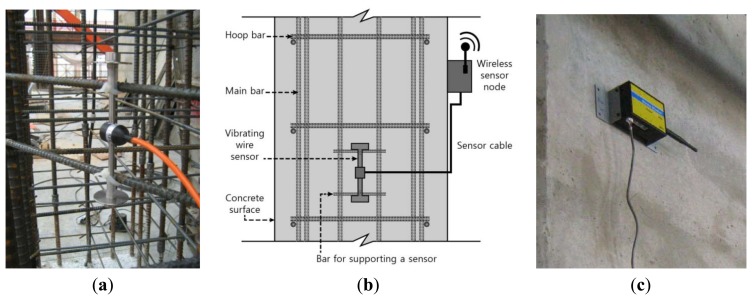
Installation of sensor and sensor node. (**a**) Installation of sensor; (**b**) Sensor and sensor node; (**c**) Installation of sensor node.

The installation of the sensor and sensor node was performed in the manner shown in Figure 8. First, a sensor is affixed to the steel reinforcement before filling the concrete in the form, and cable is organized and sealed in order to be connected to the sensor node after filling the concrete. After removal of the form, the procedure to connect the sealed cable to the sensor node is performed. The wireless nodes used in this study are basically operated with a small-capacity battery (2,700 mAh), but in actual practice, the problem of power consumption should be considered when conducting long-term automatic measurement during construction. To address this issue, sensor nodes used at this site were programmed to perform measurements for one minute and then go into sleep mode for the next 30 min. This strategy can extend the duration of the battery for approximately six months or more.

In this study, the automatic measurements were obtained using the proposed automatic sensing system over a limited period because of a client's financial demands. Thus, the measurement period and the quantities of sensing members and wireless sensing units were limited. The cost of the sensing nodes (sensor nodes, repeater nodes, and master nodes) and the monitoring software are approximately 1,400 USD and 280 USD, respectively, per month.

### 3.3. Operation of Automatic Wireless Monitoring System

**Figure 9.**
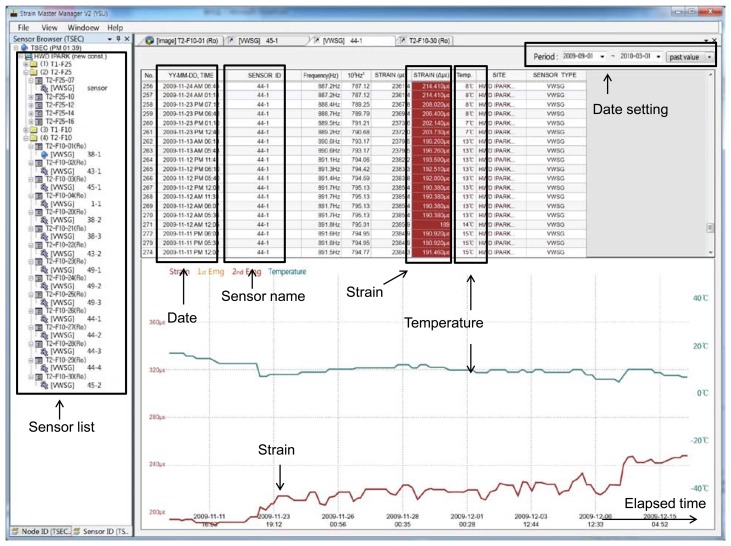
Screen capture showing measured data stored on the server through web-based managing program.

Figure 9 is a screen capture showing the actual experience of measured data stored on the server through the managing program. When the sensor is selected from the sensor list, the information of the temperature and column shortening (or axial strain) of the vertical member in a building can be checked in real-time through tables and graphs. In addition, the data can be checked over a specified range by adjusting the period of measuring, and be stored as a file, allowing easy analysis of long-term tendencies. Information on each sensor can be checked, permitting convenient comparison and analysis of measured values for each sensor.

**Figure 10.**
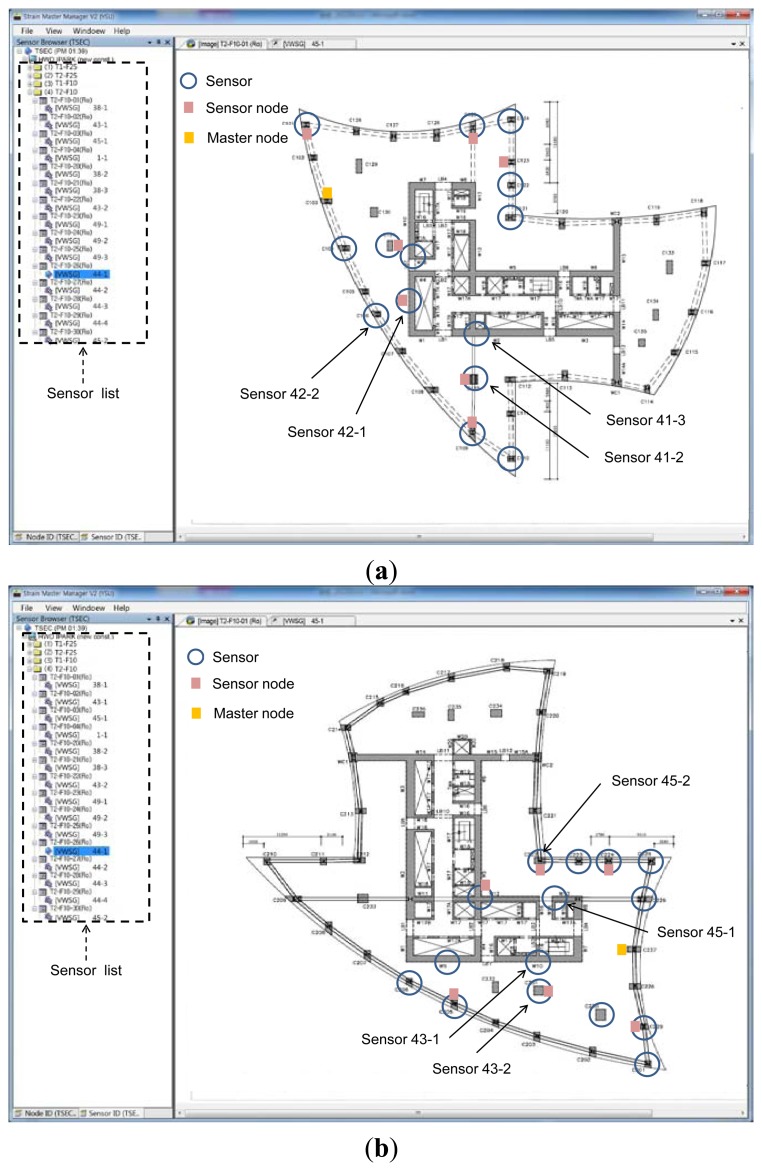
Location of sensors, sensor nodes, and master node installed at 10th floor of towers 1 and 2. (**a**) 10th floor of tower 1; (**b**) 10th floor of tower 2.

**Figure 11.**
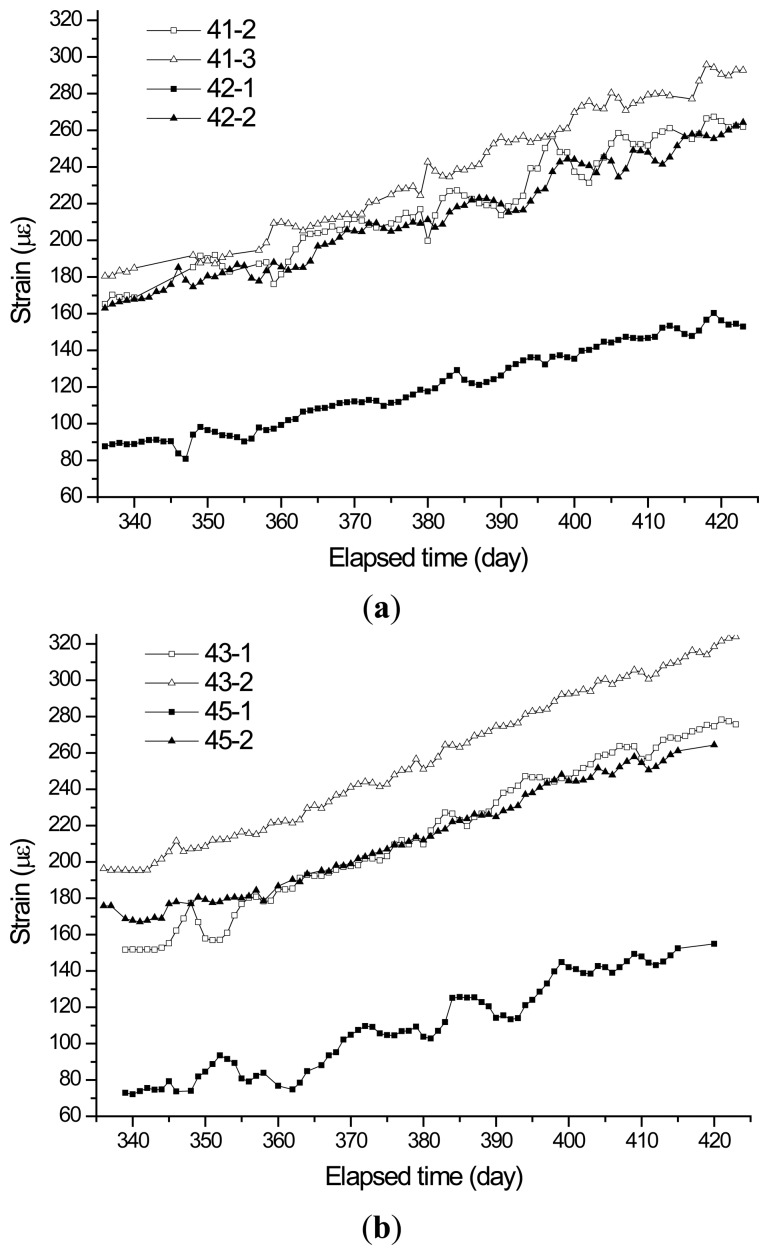
History of strains measured from sensors at the 10th floor of towers 1 and 2. (**a**) 10th floor of tower 1; (**b**) 10th floor of tower 2.

The screen captures showing the configuration of sensors and nodes installed at the 10th floor of towers 1 and 2 are shown in Figure 10. The history of vertical shortenings measured at the 10th floor of towers 1 and 2 over 88 days is shown Figure 11. The sensors installed on the 10th floor were manually measured at the beginning of the measurement. Thus, the strain data in Figure 11 did not start at zero. Figure 11 shows that the strains temporarily increase or decrease during a few days. This phenomenon can be attributed to the influence on temperature and temporary construction loads, such as construction equipment and materials. In case of this study, there was no correlation between the measured temperature and the temporary strain fluctuations.

Theoretically, there is no correlation among the sensors located in different buildings under construction because the shortening of vertical members is dependent on the respective applied loads. However, the strains measured by the sensors on the 10th floor of towers 1 and 2 increased during the elapsed time. A similar tendency for towers 1 and 2, which may be attributed to the similarity between the construction schedules of towers 1 and 2, is shown in Figure 11. A correlation can theoretically exist between the shortening of the columns on the 10th and 25th floors in the same building. However, the proposed automatic sensing system was not used to obtain simultaneous measurements in this study because of the client's financial demands. The wireless sensing units employed for the automatic measurements on the 10th floor of towers 1 and 2 were removed after 88 days of measurements and were re-installed on the 25th floor of towers 1 and 2. In this study, the correlation between the shortening of the columns on the 10th and 25th floors in the same building could not be investigated.

## 4. Discussion

**Figure 12.**
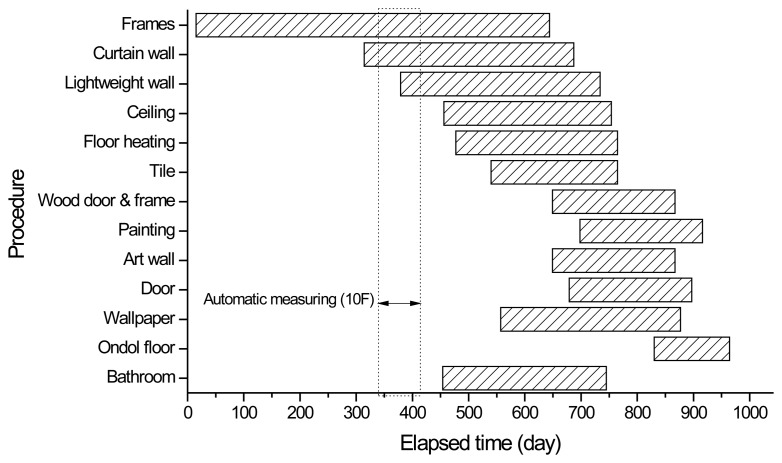
As-built construction schedule for tower 2.

During the measurement period, the processes such as construction of frames, curtain walls, and lightweight walls were performed according to the as-built construction schedule in Figure 12. The increase in loads during the construction processes could be a major factor affecting strain values measured by sensors. From Figure 11b, it is observed that the measured strain data corresponding to the magnitude of shortenings in a vertical member increased as the construction progressed.

**Figure 13.**
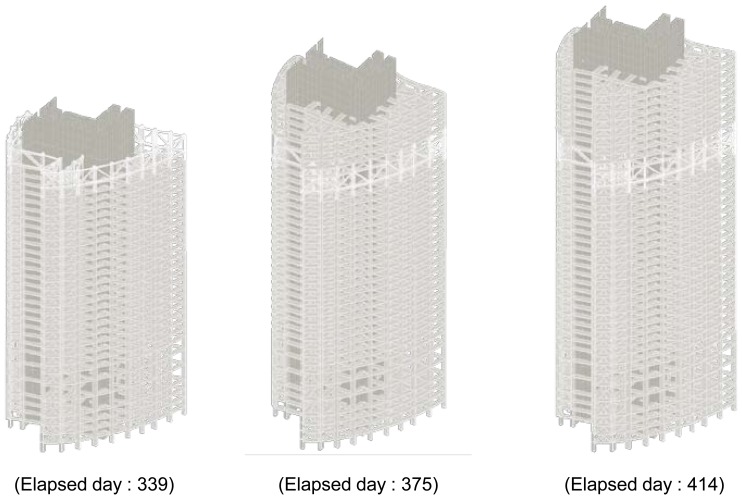
Simulation models of tower 2 for different days.

To investigate the correlation between the measured strains and the increasing weight, the strain data for elapsed days 339, 375, and 414 in Figure 11b were selected. The construction process for the three stages is shown in Figure 13. It was shown that the strains measured on the 10th floor in tower 2 are approximately 77% higher than the elastic strains, which are calculated on the structural weights, on the 10th floor in tower 2 for the period from elapsed day 339 to elapsed day 375. In addition, the strains are 69% higher than the elastic strains for the period from elapsed day 339 to elapsed day 414. The measured strains can be larger than the elastic strains because of the influences of creep, shrinkage, load uncertainty, and simulation inaccuracy. Thus, field measurements of the shortening of vertical members in high-rise buildings under construction are necessary.

**Figure 14.**
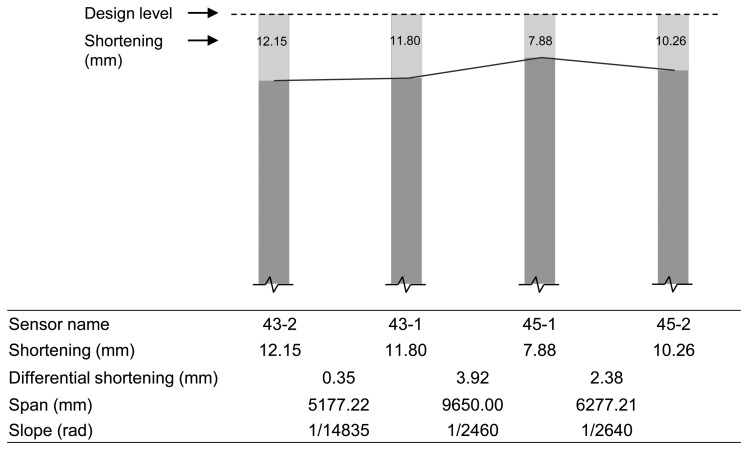
Differential shortenings for walls and columns in tower 2.

As can be seen in Figure 10b, for measurement of differential shortenings between the adjacent vertical members, sensors 43-1 and 45-1 were installed on shear walls, and sensors 43-2 and 45-2 were installed on columns adjacent to sensors 43-1 and 45-1. Using the measured strain data during the period in Figure 11b, magnitudes of accumulated axial shortenings from of the shear wall with sensor 43-1 and the column with sensor 43-2 are 12.15 mm and 11.80 mm (Figure 14), respectively. Then, the differential shortening between the wall and column is found to be 0.35 mm. Similarly, as can be seen in Figure 14, the differential shortening between the wall with sensor 45-1 and the column with sensor 45-2 is obtained. The maximum differential shortening for adjacent walls with sensors 43-1 and 45-1 is shown to be 3.92 mm (Figure 14). Thus, the magnitude of differential shortening, the degree of slab tilt due to the length changes in vertical members, can be controlled by applying compensation methods [7,8].

**Figure 15.**
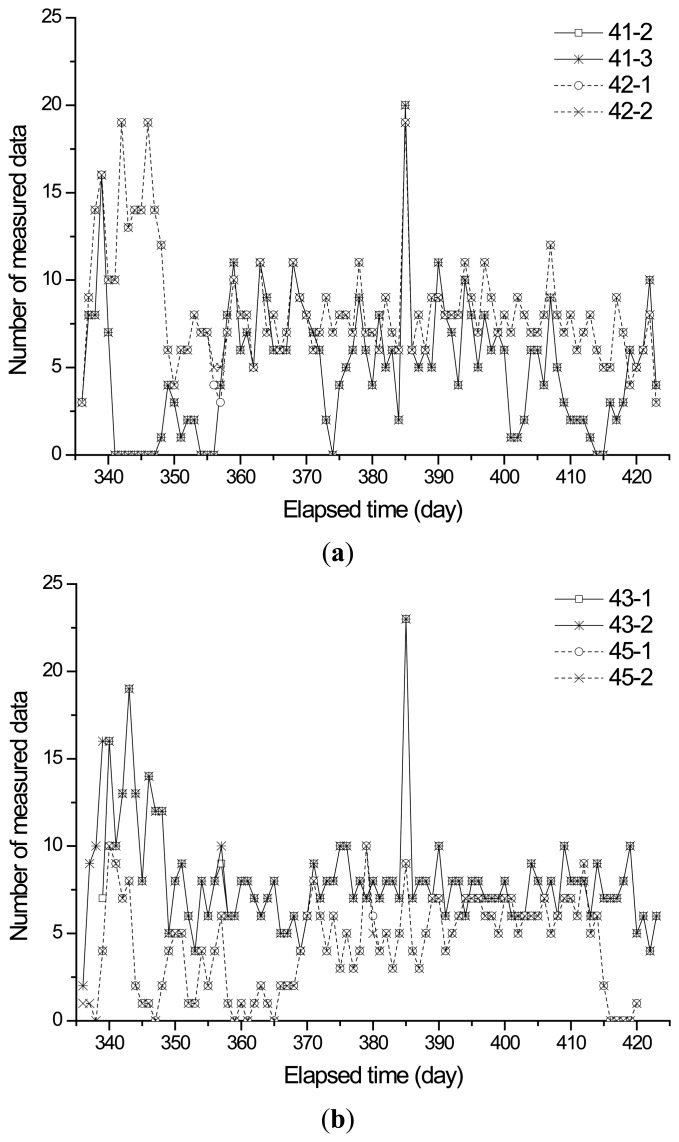
The daily data history for sensors on the 10th floor of towers 1 and 2. (**a**) 10th floor of tower 1; (**b**) 10th floor of tower 2.

Figure 15 represents the data history for the sensors on the 10th floor of towers 1 and 2. The amount of measured data fluctuated during the monitoring period because the wireless sensing units in this study employed unidirectional communication and the time synchronization was not performed during the wireless communication. The number of days for which no data are measured during the monitoring period for sensors 41-2, 41-3, 42-1, and 42-2 in tower 1 are as follows: 13 (14.77%), 13 (14.77%), 0 (0%), and 0 (0%). The number of days for which no data are measured during the monitoring period for sensors 43-1, 43-2, 45-1, and 45-2 in tower 2 are as follows: 0 (0%), 0 (0%), 8 (9.76%), and 9 (10.59%). The data loss may occur because of the sleep-action mode of the wireless sensing units, unstable power, breakdowns in the sensing system, or obstacles such as workers and construction materials.

## 5. Conclusions

In this paper, a wireless sensing system for measurement of column shortenings during construction of high-rise buildings is presented. The system automates the processes such as sensor measurements, and sending the measured data to a server through WSN which comprises the sensors, sensor nodes, master node, and repeater nodes. These nodes use the 424 MHz band with good diffraction to improve the applicability of the system in high-rise buildings and have the power-saving function of alternating between sleep mode and action mode for practical long-term automatic measurement. The data sent to the server can be monitored in real-time from any place using a web-based management program.

The proposed system was applied to the automatic monitoring of the shortening of vertical members in actual 66- and 72-floor high-rise buildings under construction. The shortening for the monitoring period during construction could be automatically obtained and promptly confirmed from any location via the Internet. This remote accessibility enables construction managers to rapidly collect and investigate a considerable amount of strain data, which can facilitate prompt actions, such as corrections for the differential shortening in construction and thus allow for more precise construction.

## Figures and Tables

**Table 1.** Number of sensors, sensor nodes, and master nodes deployed for measurement of column shortenings.

**Type**	**Tower 1**		**Tower 2**	

	**10F**	**25F**	**10F**	**25F**
Sensor	14	5	15	5
Sensor node	7	5	6	5
Master node	1	1	1	1
